# Automated generation of compartmental models via database tools for neurophysiology data management, analysis, and simulation

**DOI:** 10.1186/1471-2202-12-S1-P366

**Published:** 2011-07-18

**Authors:** Philipp Rautenberg, Andrey Sobolev, Andreas VM Herz, Thomas Wachtler

**Affiliations:** 1 Department Biology II, Ludwig-Maximilians-Universität München, 82152 Planegg-Martinsried, Germany

## 

Scientific progress depends increasingly on collaborative efforts that involve exchange of data and re-analysis of previously recorded data. A major obstacle to fully exploit the scientific potential of experimental data is the effort it takes to access both data and metadata for application of specific analysis methods, for exchange with collaborators, or for further analysis some time after the initial study was completed. To cope with these challenges and to make data analysis, re-analysis, and data sharing efficient, data together with metadata should be managed and accessed in a unified and reproducible way, so that the researcher can concentrate on the scientific questions rather than on problems of data management.

At the German Neuroinformatics Node (http://www.g-node.org), an infrastructure for cellular and systems neuroscience is being developed to improve key ingredients of neuroscientific research: data access, data storage and exchange, and data analysis [1]. One central component is a data management platform where neuroscientists can store and organize their data for sharing and analysis. Tools and interfaces for data access through a variety of applications are being developed. They enable seamless integration of data access into the laboratory workflow and efficient management and selection of data in a systematic and largely automatized fashion for data sharing and analysis.

To further demonstrate the applicability of this approach, we extended this system to generate compartmental models from the data stored in the database. Specifically, we investigated the effect of morphology on the electrophysiological properties of medial superior olive (MSO) neurons using morphology data from reconstructed neurons [2]. Simulating neurons from morphological data using compartmental models implies a highly complex parameter space, both in terms of the simulation parameters and the results. Our integrative approach employs a database to manage the data, control the simulations, and analyze the results.

Data were stored and simulations were controlled via a PostgreSQL Database. To perform simulations, we used the NEURON simulator software [3], which was integrated via Python code using plpython. Automated simulations with different morphologies were triggered within the database. We used detailed models of reconstructed MSO neurons and, for comparison, simplified models with three compartments using the same passive parameters that were estimated with the complex models. Comparison of the simulated responses of the simple models to responses of the morphologically complex models showed that the shape of the neuron affected functional properties of the neurons, but that single geometric measures like surface area are not enough to explain specific results. Therefore, we suggest to use simulated electrophysiological measures like input resistance, or effective membrane time constant to map a complex morphology to a simplified n-compartmental model.

Our study demonstrates the efficiency of a unifying approach employing a database for management of data, analysis, and simulation tools. This in turn facilitates analysis and visualization of results, which can be presented in a portable and accessible way via direct database access and web interface.
